# Serum Soluble Adhesion Molecules and Markers of Systemic Inflammation in Elderly Diabetic Patients with Mild Cognitive Impairment and Depressive Symptoms

**DOI:** 10.1155/2015/826180

**Published:** 2015-06-18

**Authors:** Malgorzata Gorska-Ciebiada, Malgorzata Saryusz-Wolska, Anna Borkowska, Maciej Ciebiada, Jerzy Loba

**Affiliations:** ^1^Department of Internal Medicine and Diabetology, Medical University of Lodz, 251 Pomorska Street, 92-213 Lodz, Poland; ^2^Department of General and Oncological Pneumology, Medical University of Lodz, 22 Kopcinskiego Street, 90-153 Lodz, Poland

## Abstract

The aim of the study was to determine the serum levels of soluble adhesion molecules and hs-CRP in elderly diabetics with mild cognitive impairment (MCI) alone or with depressive symptoms. *Methods*. 219 diabetics elders were screened for psychiatric disorders and divided: group 1, MCI without depressive mood; group 2, MCI with depressive mood; group 3, controls. Data of biochemical parameters and biomarkers were collected. *Results*. In groups 1 and 2 levels of all biomarkers were significantly higher as compared to controls. The highest level of hs-CRP and sICAM-1 was detected in group 2. SVCAM-1 and sE-selectin levels were also the highest in group 2; however they did not significantly differ as compared to group 1. MoCA score was negatively correlated with all biomarkers in group 1. The logistic regression model showed that variables which increased the likelihood of having depressive syndrome in MCI patients were older age, stroke, neuropathy, increased number of comorbidities, and higher sICAM-1 level. *Conclusions*. We first demonstrated that elderly diabetic patients with MCI, particularly those with depressive mood have higher levels of soluble adhesion molecules and markers of low-grade systemic inflammation. Coexisting depressive syndrome in patients with MCI through common inflammatory pathways may result in augmentation of psychiatric disorders.

## 1. Introduction

Mild cognitive impairment (MCI) represents the intermediate stage between normal ageing and dementia [1]. Patients diagnosed with MCI have cognitive impairment in excess of that expected for age and some limitation in complex functional activities but do not warrant a diagnosis of dementia. The prevalence of MCI has been estimated to be 14–18% for individuals aged 70 years and older [2]. MCI has been subtyped on whether memory functions are affected and if one or multiple domains of cognitive functioning are affected.

The various causes of MCI include neurodegenerative diseases, such as Alzheimer's disease, cerebrovascular disease, major psychiatric illnesses like depression, and other systemic causes [1, 3]. Patients with MCI have an increased risk of developing dementia, with an estimated annual conversion rate to dementia of 12% [1]. Type 2 diabetes (T2DM) is a risk factor for Alzheimer's disease and mild cognitive impairment. The etiology of cognitive impairment in people with T2DM is uncertain, but it is most likely multifactorial. Chronic hyperglycemia, cerebral microvascular disease, severe hypoglycemia, and increased prevalence of macrovascular disease have all been implicated but are unlikely to explain the entire effect [4]. One of the theories is that endothelial activation or inflammatory processes may be involved in the pathogenesis of MCI in diabetes [5]. Levels of circulating inflammatory markers are elevated in people with T2DM compared with nondiabetic population. Inflammatory mediators may, therefore, have a role in the accelerated development of cognitive impairment in people with diabetes either by a direct effect on the brain or through an influence on the development of atherosclerosis, vascular disease, and endothelial dysfunction [6, 7]. A lot of studies showed that depressed mood was more common in individuals with MCI than those without cognitive deficits [8, 9]. Participation of the immune system, especially excessive activation of the proinflammatory cytokines in the central nervous system in the pathogenesis of depression, has also been well documented [10].

Although there are some studies that described separately relationship of inflammation with MCI or with depressive disorders in diabetes, data about common pathogenesis included endothelial activation and low-grade inflammation in subjects with coexisting cognitive impairments and depressive disorders in elderly diabetic population are lacking. Therefore, the aim of the current study was to (1) determine the serum levels of soluble intercellular adhesion molecule 1 (sICAM-1), soluble vascular adhesion molecule 1 (sVCAM-1), and sE-selectin and high-sensitivity C-reactive protein (hs-CRP) in MCI elderly diabetics with or without coexisting depressive mood and (2) determine the predictors (including adhesion molecules and hs-CRP levels) of having depressive symptoms in patients with MCI.

## 2. Material and Methods

### 2.1. Study Population

219 participants were drawn from the study previously described elsewhere [11]. A survey was conducted among unselected elders who attended outpatient clinic belonging to the Department of Internal Medicine and Diabetology, University Hospital number 1 in Lodz, Poland. A brief screening for recruitment was conducted by the investigators to identify potential participants. We included patients aged 65 and over with diabetes type 2 diagnosed minimum 1 year earlier, subjects who had been able to understand and cooperate with study procedures. The exclusion criteria were diagnosed depression or dementia, use of possible or known cognition-impairing drugs in the previous 3 months, presence of neoplasm, constant alcohol or substance abuse, severe visual, mobility, or motor coordination impairment, history of head trauma, inflammatory or infectious brain disease, and severe neurological or psychiatric illness.

The first part of visit included a morning blood draw after a 10- to 12-hour overnight fast, blood pressure measurements, height and weight assessment, and complete physical examination. Then patients had eaten a breakfast followed by capillary glucose level measuring to ensure that participants were not hypoglycemic at the time of cognitive testing. The second part of visit took place in a private area in the clinic. Subjects completed a questionnaire describing baseline demographics and underwent cognitive testing.

### 2.2. Participant Characteristics, Clinical Evaluation, and Risk Factor Assessment

Demographic variables and possible risk factors were recorded in a standardized interview. Weight and height were measured to calculate body mass index (BMI = weight/height^2^ (Kg/m^2^)). The systolic and diastolic blood pressures (mmHg) were measured with the patient in sitting position after 5 minutes of rest. The detailed medical history of diabetes type 2 was taken and included: diabetes duration, currant treatment for diabetes and complications if present, comorbid diseases of the patient (hyperlipidemia, hypertension, cardiovascular disease, lung disease, cancer, and gastrointestinal tract diseases), and their treatment. Educational level was recorded in years of education. Diabetic vascular complications were assessed based on the existence of nephropathy, retinopathy, neuropathy, cardiovascular disease (CVD), and stroke. Hypertension was defined as either a history of hypertension or use of any antihypertensive agents, hyperlipidemia defined as use of any lipid lowering agent or an untreated serum LDL cholesterol level 2.6 mmol/L or/and triglycerides 1.7 mmol/L.

### 2.3. Blood Biochemistry

After overnight fasting, blood samples were taken by venipuncture to assess serum levels of glycosylated hemoglobin (HbA1c), fasting blood glucose (FBG), total cholesterol, triglycerides, low-density lipoprotein cholesterol (LDL-C), and high-density lipoprotein cholesterol (HDL-C). All the parameters were measured in a centralized laboratory.

### 2.4. Determination of Serum sICAM-1, sVCAM-1, sE-Selectin, and hs-CRP

The serum levels of sICAM-1, sVCAM-1, and sE-selectin were determined by Quantikine Human Immunoassay ELISA kit (R&D System, Minneapolis, USA); hs-CRP was assessed using ELISA kit (EIAab, Wuhan, China) according to the instructions of the manufacturer. Minimum detectable concentrations were 0.096 ng/mL for sICAM-1, 0.6 ng/mL for sVCAM-1, 0.009 ng/mL for sE-selectin, and 0.078 ng/mL for hs-CRP.

### 2.5. Neuropsychological Evaluations

All participants underwent the following tests: the Montreal Cognitive Assessment (MoCA) [12] to evaluate the cognitive impairment, long version of the Geriatric Depression Scale (GDS-30) [13] to assess the depressive mood, and Katz Basic Activities of Daily living (BADL) and Lawton Instrumental Activities of Daily Living (IADL) questionnaires to collect information on daily activities [14, 15]. The MoCA tests 8 cognitive domains, visual-spatial ability, attention, executive function, immediate memory, delayed memory, language, abstraction, calculation, and orientation, for a maximum total score of 30. The normal MoCA score is ≥26, with one point added if the subject has fewer than 12 years of formal education. The MoCA is better than other tools to detect MCI in the elderly patients with type 2 diabetes [16]. A diagnosis of MCI was made according to published the 2006 European Alzheimer's disease Consortium criteria [17, 18]. These are used as the currently available standard test. These criteria included absence of dementia. The cut-off points for MoCA scores (19/30) are recommended for the diagnosis of “dementia” in epidemiological studies. Patients with score of 19 and below were excluded from the study as dementia and sent to psychiatrist for further care. The criteria mentioned above included also absence of major repercussions on daily life (in our study, measured by Katz BADL and Lawton IADL).

This interview was followed by Geriatric Depression Scale (GDS) for mood assessment [13]. GDS consists of 30 items. Scores range from 0 to 9 was considered normal and 10 to 19 was considered to have depressive symptoms. Score of 20 and above was excluded from the study as severe depressive symptomatology and sent to psychiatrist for further diagnosis.

To determine the inflammatory background and endothelial activation in subjects with MCI we divided them into groups: group 1, patients with MCI without depressive mood; group 2, patients with MCI and depressive mood; group 3, controls (cognitively normal patients without depressive mood).

### 2.6. Ethics

The study was operated in accordance with the World Medical Association's Declaration of Helsinki. Each participant was assigned a number by which he/she was identified to keep his or her privacy. The approval was obtained from the independent local ethics committee of Medical University of Lodz number RNN/420/13/KB. The purpose, nature, and potential risks of the experiments were fully explained to the subjects, and all subjects gave written, informed consent at the beginning of the study. We included only patients who had been fully able to understand and cooperate with study procedures.

### 2.7. Statistical Analysis

All data are presented as means ± SD. Normality of distributions was assessed using the Shapiro-Wilk tests. From parametric methods comparison between groups was done with one-way ANOVA test followed by post hoc test for contrast. From nonparametric methods comparison between groups was done with Kruskal-Wallis ANOVA followed by post hoc test. Pearson correlation analysis for normally distributed variables and Spearman rank correlation for nonnormally distributed variables were used to assess relationships. Simple logistic regression model was done in order to select so-called independent factors which increase the selection risk of depressive symptoms in patients with MCI and then multivariable regression model in order to select the “strongest” factor from independent risk factors. To “optimize” the multivariable model, a stepwise approach was used (backward elimination with Wald criteria). Odds ratios (OR) were computed and presented with the 95% interval of confidence (CI). A *P* value of less than 0.05 was considered statistically significant. Statistica 10.0 (StatSoft, Poland, Krakow) was used for analysis.

## 3. Results

### 3.1. Clinical Characteristics

The demographic and clinical characteristics of the study group have been presented in Table 1. According to criteria mentioned above we selected 62 patients with MCI without depressive syndrome, 25 patients with MCI and depressive mood, and 132 controls. Compared with controls patients with MCI without depressive syndrome were significantly older, were less educated, and had a longer duration of diabetes; more were diagnosed with cardiovascular disease, hypertension, hyperlipidaemia, retinopathy, nephropathy, and other comorbidities. Compared with controls patients with MCI and depressive mood were older, were females, were less educated, more often smoked tobacco regularly, had higher BMI, and had a longer duration of diabetes; more were diagnosed with cardiovascular disease, stroke, neuropathy, retinopathy, hyperlipidemia, and other comorbidities.

### 3.2. The Neuropsychological Scores

MoCA score was significantly lower in subjects with cognitive impairments (groups 1 and 2). Controls had normal MoCA values. GDS-30 score was significantly higher in patients with depressive mood (group 2) and normal in other participants. Data are presented in Table 1.

### 3.3. Biochemical Parameters

The mean level of HbA1c was the highest in group 2 (MCI and depressive mood) and significantly differ from controls. The HbA1c level in group 1 (MCI without depressive mood) was also high and did not significantly differ from group 2. Controls had significantly the lowest values. The level of total cholesterol and LDL cholesterol was higher in group 2 (MCI and depressive mood) as compared to controls. The level of triglycerides was the highest and HDL was the lowest in group 2. Data are presented in Table 2.

### 3.4. Serum Levels of sICAM-1, sVCAM-1, sE-Selectin and hs-CRP

In both groups of cognitively impairment patients (group 1 and 2) the serum levels of sICAM-1, sVCAM-1, sE-selectin, and hs-CRP were significantly higher as compared to controls. The highest level of hs-CRP and sICAM-1 was detected in group 2 (MCI and depressive mood) as compared to other groups. SVCAM-1 and sE-selectin level were also the highest in group 2; however, they did not significantly differ as compared to group 1 (MCI without depressive mood). Data are presented in Table 2.

### 3.5. Correlations

We found correlations between all concentrations of adhesion molecules and inflammatory markers in groups 1 and 2. The results indicated that MoCA score was negatively correlated with all markers in group 1 (MCI without depressive mood). In group 2 (MCI and depressive mood) we found that MoCA score was negatively correlated with hs-CRP, sICAM-1, and sVCAM-1 levels, and GDS-30 score was positively correlated with all markers. In both groups (1 and 2) HbA1c level was positively correlated with all parameters. All correlations are presented in Tables 3 and 4.

### 3.6. Logistic Regression Models

Because many factors can influence the results we constructed the univariate logistic regression models and finally multivariable regression model to determine the predictors of having depressive symptoms in patients with MCI. The independent variables entered in the model at step one were demographic variables (age, gender, and education), duration of diabetes, glycaemic control (HbA1c level), cardiovascular diseases (MI, angina, and stroke), cardiovascular risk factors (BMI, smoking status, hyperlipidemia, previous HA, or use of HA drugs), microvascular complications, number of comorbid conditions, levels of hs-CRP, sICAM-1, sVCAM-1, sE-selectin, and lipids. The univariate logistic regression models revealed that variables which increased the likelihood of having depressive syndrome in patients with MCI were female gender, older age, smoking habit, higher BMI, previous stroke, neuropathy, increased number of comorbidities, and higher levels of HbA1c, hs-CRP, sICAM-1, sVCAM-1, and sE-selectin (Table 5).  Table 6 shows the results of modeling the risk of having MCI by multivariable regression. All variables presented in Table 6 were introduced to this model. The multivariable model was optimized by the stepwise approach. Older age, previous stroke, neuropathy, increased number of comorbidities, and higher sICAM-1 level are significant factors increasing the likelihood of having depressive symptoms in patients with MCI. However, the previous stroke and the presence of neuropathy are the strongest factors.

## 4. Discussion

We first demonstrated that serum levels of sICAM-1, sVCAM-1, sE-selectin, and hs-CRP in diabetic elderly patients with MCI were significantly higher as compared to controls. Moreover our results indicated that levels of these markers were negatively correlated with the MoCA score. Although several studies of the biomarkers of endothelial dysfunction have been done in patients with T2DM and most of them have demonstrated increased concentration of E-selectin, ICAM-1, and VCAM-1 data about the endothelial dysfunction in MCI are poor [19, 20]. E-selectin, ICAM-1, and VCAM-1 are important mediators for the adhesion of leukocytes to the endothelial surface and significantly related to the risk of DM complications. In agreement with our results one study had showed that markers of endothelial integrity (sVCAM-1 and sICAM-1) were specifically associated with altered cortical vasoreactivity and atrophy in multiple brain regions in both diabetic and nondiabetic participants [21]. Adhesion molecules were linked to slower walking and executive and behavioral dysfunction, which are hallmarks of behavioral decline in older adults. The authors concluded that a combination of altered vasoregulation and hyperglycemia may enhance neurotoxicity of chronic hyperglycemia in the aging diabetic brain. Another study revealed that higher sICAM-1 levels are associated with silent brain infarctions and cerebral white-matter lesions and may predict impairment in psychomotor function in type 2 diabetes [22]. The authors proposed the following mechanism: ICAM-1 expression is induced in the vascular endothelium by elevated blood glucose levels; ICAM-1 then promotes the adhesion of leucocytes, especially neutrophils, to the vascular endothelium, which causes small vessels in the brain to occlude, and leads to incidence of silent brain infarctions. The results are consistent with our findings in a multivariable logistic regression models that the previous stoke and higher sICAM-1 levels are significant factors associated with MCI and depressive symptoms in patients with T2DM.

In our study we demonstrated that MCI patients had higher levels of adhesion molecules and hs-CRP compared to controls. This is consistent with the previous research which reported that elevated circulating levels of inflammatory markers (CRP, IL-6, and TNF-*α*) were associated with poorer cognitive ability in elderly patients with T2DM [23].

However, we first discovered that the significantly highest levels of hs-CRP and adhesion molecules were observed in those MCI subjects with coexisting depressive syndrome. These observations are a novelty in literature. They suggest that low-grade inflammation and activation of endothelial adhesion molecules is possible link between these psychiatric disorders in diabetes. Hs-CR is a sensitive and dynamic marker for systemic inflammation and has been recognized as a risk factor for development and prognosis of cardiovascular diseases and for cardiovascular events in T2DM. CRP has been found in and around amyloid plaques and around small-vessel damages [5]. In agreement to our results hs-CRP could be also a marker of neuroinflammation depressed patients [24]. It has been hypothesized that explaining the association between depressive syndrome and cognitive impairment occur in patients with diabetes results from the chronic inflammatory changes that are linked to the activation of macrophages in the blood and microglia in the brain [25].

In our study the univariate logistic regression models revealed that factors variables which increased the likelihood of having been diagnosed with depressive syndrome in patients with MCI were female gender, older age, smoking habit, higher BMI, previous stroke, neuropathy, increased number of comorbidities, and higher levels of HbA1c, hs-CRP, sICAM-1, sVCAM-1, and sE-selectin. In the multivariable analysis we found the factors increasing the likelihood of having depressive syndrome in MCI patients: older age, previous stroke (the strongest factor), neuropathy, increased number of comorbidities, and higher sICAM-1. The results suggest that the higher levels of endothelial activation and inflammatory markers in MCI patients with depressive syndrome are accounted for by vascular comorbidities. Vascular pathology has been observed in patients with MCI in large epidemiologic studies [26], as well as in patients with depressive syndrome [27]. Inflammation and immune dysregulation have been critically involved in vascular disease in pathogenesis of diabetes complications [28]. Inflammation can follow, precipitate, or aggravate vascular events. Therefore, inflammation has a bidirectional role in the link between MCI, depression, and vascular pathology [5]. Thus, prospective design studies are needed to better understand the temporal associations between inflammation and these comorbidities.

Our result has also indicated that neuropathy could be a factor associated with the diagnosis of depressive syndrome in MCI subjects. It could be explained by the theory that microvascular damage characterized in T2DM is also exhibited in the central nervous system. This result increased incidence of cognitive deterioration, vascular dementia, and depression. Direct damage to small blood vessels, particularly by hyperglycemia, is manifested by endothelial dysfunction and leads to the disruption of blood-brain barrier function, which might induce neuroinflammatory reactions and neurodegeneration [29]. It is also possible that primary vascular pathology involving inflammatory cascade and blood-brain barrier breakdown will result in the leakage of serum-derived vascular components into the brain tissue and may cause brain dysfunction which, under some conditions (extent, duration, and/or location), will result in disturbed thinking processes, mood, and behavior, such as those characterizing psychiatric illnesses [30]. Although several hypothetical mechanisms have been proposed the precise mechanisms underlying T2DM-related cognitive dysfunction, development of dementia, or depressive syndrome, remains to be elucidated [31].

## 5. Limitations

This study provides important insights into the endothelial dysfunction and low-grade inflammation pathologies underlying cognitive impairment and coexisting depressive symptoms in older diabetic patients; however, it is not without limitations. First, the study was not designed as longitudinal prospective investigation. There have been few studies in which the cognitive declines in elderly diabetics were prospectively observed. However, the precise mechanisms underlying T2DM-related cognitive dysfunction or the development of dementia have not yet been elucidated. Furthermore, this investigation was limited to patients with diabetes, and, therefore, an association between endothelial and inflammatory markers with other parameters in subjects without diabetes should also be assessed.

## 6. Conclusions

In our study we demonstrated that elderly diabetic patients with mild cognitive impairment, particularly those with coexisting depressive mood, have higher levels of soluble adhesion molecules and markers of low-grade systemic inflammation. Coexisting depressive syndrome in patients with cognitive impairment through common inflammatory pathways may result in augmentation of psychiatric disorders. Previous CVD, neuropathy, increased number of comorbidities, and higher sICAM-1 level are the factors increasing the likelihood of having depressive syndrome in MCI patients. Further longitudinal larger studies that measure a range of biomarkers at different stages of decline are needed in order to better understand the role of inflammation on cognition and its progression to dementia.

## Figures and Tables

**Table 1:** Demographic and clinical characteristics of type 2 diabetic elderly patients.

	All subjects	Group 1	Group 2	Group 3
Number	219	62	25	132
Age (years)	73.8 ± 5.1	74.7 ± 3.9^*∗*^	78.0 ± 5.3^†^	72.5 ± 4.9^‡^
Sex, female	103 (47%)	32 (51.6%)^*∗*^	21 (84%)^†^	50 (37.8%)
Years of education	11.1 ± 2.4	9.8 ± 1.9	9.5 ± 1.7^†^	12.1 ± 2.2^‡^
Smoked tobacco regularly	60 (27.4%)	12 (19.4%)^*∗*^	14 (56%)^†^	34 (25.7%)
BMI (kg/m^2^)	29.3 ± 3.4	29.8 ± 3.5	31.8 ± 3.2^†^	28.6 ± 3.1
Systolic BP (mm Hg)	136.4 ± 15.6	136.9 ± 16.1	135.4 ± 17.8	136.4 ± 15.2
Diastolic BP (mm Hg)	75.2 ± 8.1	75.8 ± 8.1	74.9 ± 8.2	75.3 ± 8.1
FBG (mmol/L)	129.3 ± 25.8	129.6 ± 26.3	130.4 ± 29.9	128.9 ± 25.1
Duration of DM2 (years)	8.36 ± 6.1	10.63 ± 6.2	12.8 ± 6.23^†^	6.45 ± 5.07^‡^
Previous CVD	99 (45.2%)	48 (77.4%)	23 (92%)^†^	28 (21.2%)^‡^
Stroke	12 (5.5%)	2 (3.2%)^*∗*^	5 (20%)^†^	5 (3.78%)
Previous HA/use of HA drugs	174 (79.5%)	60 (96.7%)	21 (84%)	93 (70.4%)^‡^
Hyperlipidemia	166 (75.8%)	56 (90.3%)	24 (96%)^†^	86 (65.15%)^‡^
Retinopathy	104 (47.5%)	43 (69.4%)	18 (72%)^†^	43 (32.6%)^‡^
Nephropathy	81 (36.9%)	32 (51.6%)	11 (44%)	38 (28.8%)^‡^
Neuropathy	33 (15.1%)	10 (16.1%)^*∗*^	10 (40%)^†^	13 (9.8%)
Comorbidity (*n*)	4.49 ± 3.2	6.3 ± 3.06^*∗*^	9 ± 2.82^†^	2.8 ± 1.8^‡^
MoCA score	25.01 ± 3.1	21.5 ± 1.5	21.8 ± 1.7^†^	27.3 ± 1.2^‡^
GDS-30 score	4.46 ± 4.9	3.6 ± 2.7^*∗*^	16 ± 2.7^†^	2.7 ± 2.6

Group 1, MCI without depressive syndrome; group 2, MCI with depressive syndrome; group 3, controls.

Values are expressed by mean ± SD or frequency. ^*∗*^indicates difference between group 1 and group 2; ^†^group 2 and group 3; ^‡^group 1 and group 3, respectively. The ANOVA test followed by post hoc test was used to test for significant differences. A *P* value of less than 0.05 was considered statistically significant.

DM2: diabetes type 2, CVD: cardiovascular disease, HA: hypertension, BMI: body mass index, BP: blood pressure, FBG: fasting blood glucose, MoCA: Montreal Cognitive Assessment, and GDS: Geriatric Depression Scale.

**Table 2:** Inflammatory markers and biochemical characteristics of type 2 diabetic elderly patients.

	All subjects (*n* = 219)	Group 1 (*n* = 62)	Group 2 (*n* = 25)	Group 3 (*n* = 132)	*P* value
hs-CRP (ng/mL)	1.55 ± 1.3	2.39 ± 1.4^*∗*^	3.11 ± 1.5^†^	0.86 ± 0.67^‡^	*P* < 0.001
sICAM-1 (ng/mL)	224.3 ± 58.7	260.3 ± 35.67^*∗*^	315.1 ± 65.9^†^	190.1 ± 31.52^‡^	*P* < 0.001
sVCAM-1 (ng/mL)	816.1 ± 233.6	937.3 ± 161.7	1023 ± 202.2^†^	720 ± 216.2^‡^	*P* < 0.001
sE-selectin (ng/mL)	57.68 ± 11.9	61.61 ± 10.42	67.08 ± 10.5^†^	54.06 ± 11.29^‡^	*P* < 0.001
HbA1c (%)	7.29 ± 0.7	7.62 ± 0.69	8.02 ± 0.68^†^	7 ± 0.5^‡^	*P* < 0.001
CHOL (mmol/L)	9.87 ± 1.9	10.13 ± 2.2	10.75 ± 2.1^†^	9.58 ± 1.76	*P* = 0.011
LDL (mmol/L)	5.79 ± 1.57	5.79 ± 1.49	6.54 ± 1.89^†^	5.64 ± 1.5	*P* = 0.031
TG (mmol/L)	9.77 ± 2.28	10.29 ± 2.66	11.33 ± 2.63^†^	9.24 ± 1.79^‡^	*P* < 0.001
HDL (mmol/L)	2.52 ± 0.54	2.36 ± 0.64	2.15 ± 0.45^†^	2.66 ± 0.44^‡^	*P* < 0.001

Group 1, MCI without depressive syndrome; group 2, MCI with depressive syndrome; group 3, controls.

Values are expressed by mean ± SD or frequency. ^*∗*^indicates difference between group 1 and group 2; ^†^group 2 and group 3; ^‡^group 1 and group 3, respectively. The ANOVA test followed by post hoc test was used to test for significant differences. *P* values are presented in the table.

hs-CRP: high-sensitivity C-reactive protein, sICAM-1: soluble intercellular adhesion molecule 1, sVCAM-1: soluble vascular adhesion molecule 1, HbA1c: glycosylated hemoglobin, LDL: low-density lipoprotein, HDL: high-density lipoprotein, TG: triglycerides, and CHOL: total cholesterol.

**Table 3:** Correlations coefficients among serum levels of sICAM-1, sVCAM-1, sE-Selectin, hs-CRP, HbA1c, lipids, BMI, and MoCA score in group 1 (MCI without depressive syndrome).

	sICAM-1 (ng/mL)	sVCAM-1 (ng/mL)	sE-selectin (ng/mL)	MoCA score	HbA1c (%)	CHOL (mmol/L)	LDL (mmol/L)	HDL (mmol/L)	TG (mmol/L)	BMI (kg/m^2^)
hs-CRP (ng/mL)	0.47^*∗∗∗*^	0.34^*∗∗*^	0.02	−0.58^*∗∗∗*^	0.56^*∗∗∗*^	0.25^*∗*^	0.18	−0.02	0.03	0.32^*∗*^
sICAM-1 (ng/mL)		0.69^*∗∗∗*^	0.34^*∗∗*^	−0.66^*∗∗∗*^	0.64^*∗∗∗*^	0.24	0.24	−0.37^*∗∗*^	0.19	0.14
sVCAM-1		1	0.29^*∗*^	−0.44^*∗∗∗*^	0.55^*∗∗∗*^	0.24	0.22	−0.21	0.12	0.03
E-selectin			1	−0.41^*∗∗*^	0.25^*∗∗∗*^	0.13	0.07	0.04	0.11	0.03
MoCA				1	−0.63^*∗∗∗*^	−0.13	−0.14	0.05	−0.06	−0.29^*∗*^

^*∗*^ *P* < 0.05, ^*∗∗*^ *P* < 0.01, and  ^*∗∗∗*^ *P* < 0.001; hs-CRP: high-sensitivity C-reactive protein, sICAM-1: soluble intercellular adhesion molecule 1, sVCAM-1: soluble vascular adhesion molecule 1, BMI: body mass index, MoCA: Montreal Cognitive Assessment, HbA1c: glycosylated hemoglobin, LDL: low-density lipoprotein, HDL: high-density lipoprotein, TG: triglycerides, and CHOL: total cholesterol.

**Table 4:** Correlations coefficients among serum levels of ICAM-1, VCAM-1, sE-selectin, hs-CRP HbA1c, lipids, BMI, MoCA, and GDS-30 score in group 2 (MCI with depressive syndrome).

	sICAM-1 (ng/mL)	sVCAM-1 (ng/mL)	sE-selectin (ng/mL)	GDS-30 score	MoCA score	HbA1c (%)	CHOL (mmol/L)	LDL (mmol/L)	HDL (mmol/L)	TG (mmol/L)	BMI (kg/m^2^)
hs-CRP (ng/mL)	0.49^*∗*^	0.58^*∗∗*^	0.33	0.54^*∗∗*^	−0.61^*∗∗∗*^	0.85^*∗∗∗*^	0.45^*∗*^	0.21	−0.44^*∗*^	0.3	0.18
sICAM-1 (ng/mL)		0.53^*∗∗*^	0.18	0.39^*∗*^	−0.51^*∗*^	0.57^*∗∗*^	0.11	0.12	−0.21	0.1	0.18
sVCAM-1		1	0.42^*∗*^	0.28	−0.44^*∗*^	0.61^*∗∗*^	0.07	0.12	−0.44^*∗*^	0.32	0.21
sE-selectin			1	0.49^*∗*^	−0.37	0.43^*∗*^	0.01	0.12	−0.34	0.21	0.26
GDS				1	−0.63^*∗∗*^	0.57^*∗∗*^	0.38	0.25	−0.23	−0.01	0.43^*∗*^
MoCA					1	−0.55^*∗∗*^	−0.19	0.11	0.15	0.09	−0.32

^*∗*^ *P* < 0.05, ^*∗∗*^ *P* < 0.01, and ^*∗∗∗*^ *P* < 0.001; hs-CRP: high-sensitivity C-reactive protein, sICAM-1: soluble intercellular adhesion molecule 1, sVCAM-1: soluble vascular adhesion molecule 1, BMI: body mass index, MoCA: Montreal Cognitive Assessment, GDS: Geriatric Depression Scale, HbA1c: glycosylated hemoglobin, LDL: low-density lipoprotein, HDL: high-density lipoprotein, TG: triglycerides, and CHOL: total cholesterol.

**Table 5:** Assessment results of the risk of having depressive syndrome in a simple logistic regression model in the patients with MCI.

Variables analyzed	*β*	SE of *β*	*P* value	OR	95% CI
Age (years)^*∗*^	0.17	0.06	0.004	1.19	1.05–1.34
Sex: female^*∗*^	0.79	0.3	0.008	2.22	1.23–4.00
Years of education	−0.08	0.13	0.52	0.92	0.71–1.18
Smoked tobacco regularly^*∗*^	0.83	0.25	0.001	2.3	1.39–3.82
BMI (kg/m^2^)^*∗*^	0.16	0.07	0.02	1.17	1.02–1.35
Duration of DM2 (years)	0.05	0.03	0.15	1.05	0.98–1.13
Previous CVD	0.61	0.39	0.12	1.83	0.83–4.00
Previous stroke^*∗*^	1.1	0.43	0.02	2.74	1.16–6.45
Previous HA or use of HA drugs	0.87	0.45	0.053	2.3	0.98–5.78
Hyperlipidemia	0.47	0.05	0.39	1.6	0.54–4.74
Retinopathy	0.06	0.26	0.81	1.06	0.63–1.78
Nephropathy	0.15	0.23	0.51	0.85	0.53–1.36
Neuropathy^*∗*^	0.62	0.26	0.02	1.86	1.1–3.14
Comorbidity (*n*)^*∗*^	0.31	0.09	0.001	1.36	1.13–1.64
hs-CRP (ng/mL)^*∗*^	0.37	0.17	0.04	1.45	1.01–2.05
sICAM-1 (ng/mL)^*∗*^	0.02	0.006	*P* < 0.001	1.03	1.01–1.04
sVCAM-1 (ng/mL)^*∗*^	0.003	0.001	0.045	1.00	1.00–1.01
sE-selectin (ng/mL)^*∗*^	0.05	0.02	0.034	1.05	1.00–1.1
HbA1c (%)^*∗*^	0.95	0.39	0.02	2.47	1.14–5.34
CHOL (mmol/L)	0.007	0.006	0.23	1.01	0.99–1.02
LDL (mmol/L)	0.015	0.008	0.06	1.02	0.99–1.03
TG (mmol/L)	0.009	0.006	0.11	1.01	0.99–1.02
HDL (mmol/L)	−0.39	0.02	0.14	0.96	0.91–1.01

*β*: regression coefficient; CI: confidence interval for odds ratio; OR: odds ratio; SE: standard error; ^*∗*^significance, *P* < 0.05.

**Table 6:** Assessment results of the risk of having depressive syndrome in a multivariable logistic regression model in the patients with MCI.

Variables analyzed	*β*	SE of *β*	*P* value	OR	95% CI
Age (years)^*∗*^	0.2	0.08	0.014	1.22	1.04–1.43
Previous stroke^*∗*^	1.32	0.66	0.045	3.76	1.03–13.75
sICAM-1 (ng/mL)^*∗*^	0.02	0.008	0.002	1.02	1.1–1.04
Comorbidity (*n*)^*∗*^	0.23	0.11	0.043	1.25	1.01–1.56
Neuropathy^*∗*^	1.25	0.43	0.004	3.49	1.49–8.15

*β*: regression coefficient; CI: confidence interval for odds ratio; OR: odds ratio; SE: standard error; ^*∗*^significance, *P* < 0.05.
